# Vaginal laxity: which measure of levator ani distensibility is most predictive?

**DOI:** 10.1002/uog.21873

**Published:** 2020-05-01

**Authors:** C. Manzini, T. Friedman, F. Turel, H. P. Dietz

**Affiliations:** ^1^ Department of Obstetrics and Gynecology University Medical Centre Utrecht Utrecht The Netherlands; ^2^ University of Sydney Sydney Australia

**Keywords:** 3D/4D ultrasound, pelvic floor, translabial ultrasound, vaginal laxity

## Abstract

**Objective:**

To assess the predictive value of measures of levator hiatal distension at rest and on maximum Valsalva maneuver for symptoms of vaginal laxity.

**Methods:**

This was a retrospective study of women seen at a tertiary urogynecological unit. All women underwent a standardized interview, clinical examination and four‐dimensional translabial ultrasound examination. Area, anteroposterior diameter (APD) and coronal diameter (CD) of the levator hiatus were measured at rest and on maximum Valsalva maneuver in the plane of minimal hiatal dimensions using the rendered volume technique, by an operator blinded to all clinical data. The association between levator hiatal measurements and vaginal laxity was assessed, and receiver‐operating‐characteristics (ROC)‐curve analysis was used to determine their predictive value.

**Results:**

Data from 490 patients were analyzed. Mean age was 58 (range, 18–88) years, and vaginal laxity was reported by 111 (23%) women. Measurements obtained on maximum Valsalva were significantly larger in women who reported vaginal laxity than in those who did not, with mean levator hiatal area, APD and CD of 30.45 ± 8.74 cm^2^, 7.24 ± 1.16 cm and 5.60 ± 0.89 cm, respectively, in the vaginal‐laxity group, compared with 24.84 ± 8.63 cm^2^, 6.64 ± 1.22 cm and 5.01 ± 0.97 cm in the no‐laxity group (*P* < 0.001 for all). Measurements obtained at rest were not significantly different between the groups. Multiple logistic regression analysis controlling for age, body mass index, vaginal parity and levator avulsion confirmed these results. The best regression model for the prediction of vaginal laxity included age, vaginal parity and levator hiatal area on maximum Valsalva. ROC‐curve analysis of levator hiatal measurements on maximum Valsalva in the prediction of vaginal laxity demonstrated areas under the curve of 0.68 (95% CI, 0.63–0.73) for area, 0.63 (95% CI, 0.57–0.68) for APD and 0.68 (95% CI, 0.62–0.73) for CD.

**Conclusions:**

Levator hiatal area on maximum Valsalva seems to be the measure of levator ani distensibility that is most predictive of symptoms of vaginal laxity. Copyright © 2019 ISUOG. Published by John Wiley & Sons Ltd.

**CONTRIBUTION**

*What are the novel findings of this work?*

Our results show a clear, statistically significant increase in measures of pelvic floor distensibility in women complaining of vaginal laxity, supporting the growing evidence of an association between vaginal laxity and pelvic floor hyperdistensibility, and contributing to a method for objectively defining this condition.

*What are the clinical implications of this work?*

Since vaginal laxity is likely to be under‐reported by patients, and given its association with pelvic floor hyperdistensibility, gynecologists should be sure to investigate sexual function, especially in women with a clinically wide hiatus or hiatal ‘ballooning’ on translabial ultrasound.

## INTRODUCTION

Vaginal laxity is a poorly investigated symptom of pelvic floor dysfunction. It has been identified only recently as a symptom of sexual dysfunction that is related to pelvic organ prolapse (POP), and has been defined as a complaint of excessive vaginal looseness^1^. It is experienced mostly as reduced vaginal sensation during sexual intercourse, carrying physical and emotional consequences^2^.

There is no objective, standardized diagnostic test for vaginal laxity^3^. In the literature, it has been defined as a self‐reported symptom that can be elicited by interviews or questionnaires^1^, ^2^, ^3^, ^4^, ^5^, ^6^, ^7^, ^8^. In a survey of urogynecologists, 83% of 563 respondents stated that vaginal laxity is under‐reported by their patients^4^; this implies that there is a high number of affected women who are undiagnosed. Some studies have investigated its presence among women attending a urogynecology clinic, documenting a prevalence of 24–38%^5^, ^6^, but no information is available regarding its incidence in the general population.

There is a consensus on the association of vaginal laxity with pregnancy and childbirth^7^, ^8^, ^9^. However, its pathophysiology is not completely understood. While one proposed mechanism involves overstretching of the vaginal walls and the introitus during vaginal delivery, an alternative pathophysiological process may be related to an increase in levator ani hiatal dimensions resulting from trauma to the levator ani muscle via frank avulsion (macrotrauma) or overdistension (microtrauma)^10^, ^11^.

An association between vaginal laxity and measures of levator ani hyperdistensibility (genital hiatus (Gh) plus perineal body (Pb), and levator hiatal area on Valsalva maneuver) has been demonstrated in a previous study^5^. On the basis of these results, we designed the current retrospective study to assess the predictive value of different measures of levator hiatal distension, obtained at rest and on maximum Valsalva maneuver, for symptoms of vaginal laxity and symptom bother, in order to identify which measure of levator ani distensibility is most predictive of the symptoms of vaginal laxity.

## METHODS

This was a retrospective study based on archived datasets of women with symptoms of pelvic floor and lower urinary tract dysfunction, examined between 26 May 2016 and 20 July 2017 at a tertiary urogynecological center. All patients underwent a locally developed standardized interview, clinical examination and four‐dimensional translabial ultrasound (4D‐TLUS) examination. During the interview, symptoms of vaginal laxity were elicited by asking: ‘Have you noticed vaginal laxity or looseness?’, and subjective vaginal laxity symptom bother was assessed using a visual analog scale from 0 (no bother at all) to 10 (worst conceivable bother). Clinically significant POP was defined as stage ≥ 2 in the anterior and posterior compartments, and ≥ 1 centrally, on the POP quantification system^12^.

4D‐TLUS was performed using a Voluson 730 Expert or Voluson S6 machine with 4–8‐MHz curved array volume transducers (GE Healthcare, Zipf, Austria) with the woman in the supine position, after emptying her bladder, at rest and on maximum Valsalva maneuver. Ultrasound volumes were assessed by the first author (C.M.) at a later date using proprietary software (4D view v. 10, GE Healthcare), blinded to all other data.

**Figure 1:**
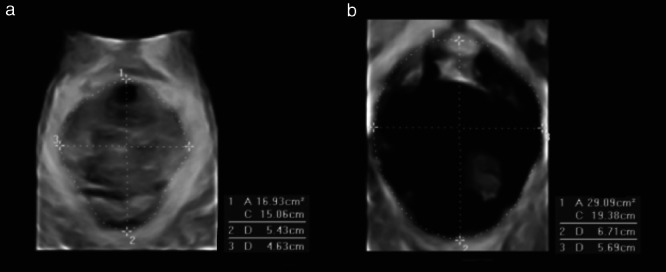
Ultrasound images of levator hiatus at rest (a) and on maximum Valsalva maneuver (b), on which area, anteroposterior diameter and coronal diameter were measured.

As described previously^13^, levator hiatal area, anteroposterior diameter (APD) and coronal diameter (CD) at rest and on maximum Valsalva maneuver were measured in the plane of minimal hiatal dimensions using the rendered volume technique (Figure 1). The change in those parameters from rest to maximum Valsalva (delta value) was calculated using the formula: 100(X_Valsalva_ – X_rest_)/X_Valsalva_. Significant POP on TLUS was defined as a bladder and rectal ampulla descent to ≥ 10 mm and ≥ 15 mm below the pubic symphysis, respectively, and descent of the uterus to ≤ 15 mm above the pubic symphysis.

A test–retest series for all investigated measures of levator ani distensibility was undertaken by two observers (C.M., F.T.). Interobserver reliability was determined using the intraclass correlation coefficient (ICC) (single measure, absolute agreement).

Statistical analysis was carried out using IBM SPSS software v. 22 (IBM Corp., Armonk, NY, USA). Mean values were compared using an independent samples *t*‐test. Logistic regression and Pearson's correlation were used to assess the association between levator hiatal distension parameters and vaginal laxity and symptom bother, respectively. To identify the best regression model for the prediction of vaginal laxity, a forward selection procedure was used, in which explanatory variables were retained or removed from the model based on the likelihood ratio test, using *P* < 0.05 as the entry criterion and *P* > 0.1 as the exit criterion. As there were more than three times the number of women in the no‐laxity group than in the vaginal‐laxity group, in order to avoid class imbalance, we included all patients with vaginal laxity as well as a random selection of patients from the no‐laxity group, so that the two groups were equal in size. Parameters tested as explanatory variables were age, body mass index (BMI), vaginal parity, levator avulsion and levator hiatal area, APD and CD on maximum Valsalva. Receiver‐operating‐characteristics (ROC) curves were constructed for measures of levator hiatal distension on maximum Valsalva in the prediction of vaginal laxity.

This retrospective study was approved by the local Human Research Ethics Committee (NBMLHD HREC 13‐70).

## RESULTS

Of the 515 women seen during the study period, 25 were excluded owing to missing clinical or ultrasound volume data, leaving 490 patients for the analysis. Mean age was 58 ± 13 (range, 18–88) years, and mean BMI was 30 ± 7 (range, 15–68) kg/m^2^. There were 318 (65%) postmenopausal women, of whom 13% (40/318) were on hormone replacement therapy. 434 patients (89%) had a history of vaginal delivery, of whom 32% (138/434) had a history of forceps delivery. Of the women, 151 (31%) had previously undergone a hysterectomy, 80 (16%) had undergone prolapse surgery and 58 (12%) had a history of incontinence surgery. Symptoms of prolapse were reported by 240 patients (49%), those of stress incontinence by 351 (72%), those of urge incontinence by 360 (73%), those of urinary frequency by 148 (30%), those of nocturia by 187 (38%) and those of vaginal laxity by 111 (23%), with a mean vaginal‐laxity symptom bother score of 5.8/10. Clinically significant POP was detected in 360 (74%) patients, including 266 (54%) cases of cystocele, 121 of uterine prolapse (36% of 339 women who did not have a hysterectomy) and 281 (57%) of posterior compartment prolapse. On TLUS, significant cystocele, uterine prolapse, rectocele and enterocele were identified in 183 (37%), 112/339 (33%), 204 (42%) and 36 (7%) cases, respectively (Table 1). There were 110 patients (22%) with an avulsion and 253 (52%) showed abnormal distensibility of the levator hiatus (hiatal area on maximum Valsalva of ≥ 25 cm^2^).

**Table 1:** Demographics, symptoms at presentation and pelvic organ prolapse (POP) assessment of 490 women with pelvic floor and lower urinary tract dysfunction examined at a tertiary urogynecological unit

Variable	Value
Age (years)	58 ± 13 (18–88)
BMI (kg/m^2^)	30 ± 7 (15–68)
Postmenopausal	318 (65)
Parous (vaginal)	434 (89)
Previous forceps delivery	138 (28)
Previous hysterectomy	151 (31)
Previous POP surgery	80 (16)
Previous incontinence surgery	58 (12)
Prolapse symptoms	240 (49)
Vaginal laxity	111 (23)
POP‐Q assessment	
Significant cystocele	266 (54)
Significant uterine prolapse*	121/339 (36)
Significant posterior compartment prolapse	281 (57)
TLUS assessment	
Significant cystocele	183 (37)
Significant uterine prolapse*	112/339 (33)
Significant rectocele	204 (42)
Enterocele	36 (7)

Data are given as mean ± SD (range), *n* (%) or *n*/*N* (%).

* Measured for 339 patients who did not have hysterectomy.

BMI, body mass index; POP‐Q, POP quantification system; TLUS, translabial ultrasound.

In a test–retest series of 20 patients, measurements of levator hiatal area, APD and CD demonstrated good to excellent interobserver repeatability, with respective ICC values of 0.86 (95% CI, 0.57–0.95), 0.85 (95% CI, 0.55–0.94) and 0.61 (95% CI, 0.25–0.83) for measurements obtained on maximum Valsalva, and 0.79 (95% CI, 0.53–0.91), 0.74 (95% CI, 0.45–0.89) and 0.86 (95% CI, 0.69–0.94) for those obtained at rest.

Table 2 provides mean values of hiatal area, APD and CD obtained at rest and on maximum Valsalva in women who reported vaginal laxity and those who did not. Measurements obtained on maximum Valsalva were significantly different between groups, while those obtained at rest were not. On univariate analysis, measurements of levator hiatal distension on maximum Valsalva had a strong significant association with vaginal laxity (*P* < 0.001), and a multiple logistic regression analysis controlling for age, BMI, vaginal parity and levator avulsion confirmed these results.

**Table 2:** Measurements of levator hiatal area, anteroposterior diameter (APD) and coronal diameter (CD) obtained at rest and on maximum Valsalva maneuver in 490 women with symptoms of pelvic floor and lower urinary tract dysfunction, according to whether they reported vaginal laxity

Levator hiatalvariable	No vaginal laxity (*n* = 379)	Vaginal laxity (*n* = 111)	*P* *
Hiatal area (cm^2^)			
At rest	15.49 ± 4.47	16.29 ± 3.78	0.088
On Valsalva	24.84 ± 8.63	30.45 ± 8.74	< 0.001
Delta (%)	33.68 ± 18.09	44.02 ± 14.19	< 0.001
APD (cm)			
At rest	5.64 ± 0.87	5.79 ± 0.74	0.117
On Valsalva	6.64 ± 1.22	7.24 ± 1.16	< 0.001
Delta (%)	13.71 ± 11.72	18.84 ± 11.62	< 0.001
CD (cm)			
At rest	4.09 ± 0.69	4.24 ± 0.76	0.053
On Valsalva	5.01 ± 0.97	5.60 ± 0.89	< 0.001
Delta (%)	16.86 ± 13.72	23.54 ± 11.81	< 0.001

Data are given as mean ± SD.

* Independent samples *t*‐test.

The best regression model for the prediction of vaginal laxity included as explanatory variables age, vaginal parity and levator hiatal area on maximum Valsalva, classifying correctly 67% of cases compared with 50% using the null model (model without explanatory variables in which all cases are assigned to the no‐laxity group). Levator hiatal APD and CD on maximum Valsalva were not included in the final model, which confirms that levator hiatal area on maximum Valsalva is the measure of levator ani distensibility with the best predictive value for vaginal laxity.

Table 3 shows the correlation between measurements of levator hiatal distension and vaginal laxity symptom bother score. All measurements on maximum Valsalva and CD at rest were correlated with symptom bother score (*P* < 0.001 and *P* = 0.035, respectively).

**Table 3:** Correlation of measurements of levator hiatal area, anteroposterior diameter (APD) and coronal diameter (CD) obtained at rest and on maximum Valsalva maneuver, with vaginal laxity symptom bother score in 471 women*  with symptoms of pelvic floor and lower urinary tract dysfunction

Levator hiatal variable	*r*	*P*
Area		
At rest	0.082	0.076
On Valsalva	0.232	< 0.001
APD		
At rest	0.072	0.117
On Valsalva	0.185	< 0.001
CD		
At rest	0.097	0.035
On Valsalva	0.228	< 0.001

Analysis performed using Pearson's correlation.

* Symptom bother scores missing for 19 women.

ROC curve analysis of levator hiatal measurements obtained on maximum Valsalva in the prediction of vaginal laxity demonstrated areas under the ROC curve of 0.68 (95% CI, 0.63–0.73) for area, 0.63 (95% CI, 0.57–0.68) for APD and 0.68 (95% CI, 0.62–0.73) for CD. ROC curves for the delta values (change from rest to Valsalva) of levator hiatal measurements did not significantly increase the predictive power; hence, calculating delta values is not likely to be clinically useful.

For levator hiatal area on maximum Valsalva, the best cut‐off for the prediction of vaginal laxity was 26 cm^2^, with sensitivity of 0.64 and specificity of 0.60.

## DISCUSSION

Vaginal laxity is a poorly investigated and probably under‐reported symptom of pelvic floor dysfunction^4^, which is defined as a complaint of excessive vaginal looseness^1^, meaning that the vagina is not firmly held in place, often with consequent reduced vaginal sensation during sexual intercourse^2^. As there is no objective, standardized diagnostic test for vaginal laxity^3^, it is a self‐reported condition and, having been neglected for a long time, it clearly deserves further study.

One of the most important results of the current study is the clear, statistically significant increase in measurements of pelvic floor distensibility in women complaining of vaginal laxity. Our findings support the growing evidence of an association between vaginal laxity and pelvic floor hyperdistensibility^5^, and contribute to a method for objectively defining this condition. Of note, for levator hiatal area on maximum Valsalva, the best cut‐off for the prediction of vaginal laxity was 26 cm^2^, confirming the standard definition of ‘ballooning’ or excessive distensibility of the levator hiatus, which is ≥ 25 cm^2^ 14.

The best regression model for the prediction of vaginal laxity included as explanatory variables age, vaginal parity and levator hiatal area on maximum Valsalva, which confirms that levator hiatal area on Valsalva is the measure of levator ani distensibility with the best predictive value for vaginal laxity, given that levator hiatal APD and CD on maximum Valsalva were not included in the final model. As suggested previously^5^, the association with age implies that this complaint may primarily affect younger women, probably because it is commonly perceived during sexual intercourse. The association with vaginal parity confirms the role of vaginal delivery in the pathophysiology of the symptoms.

Interestingly, women who reported vaginal laxity had statistically significantly larger hiatal dimensions on maximum Valsalva, but not at rest. These results confirm a previous finding of an association between vaginal laxity and levator ani hiatal area on maximum Valsalva^5^. The fact that measurements obtained at rest are not significantly different between the two groups may seem surprising. However, measurements obtained on maximum Valsalva are likely to be more indicative of the biomechanical properties of the muscle than those obtained at rest and with the woman in the supine position, i.e. without loading of the structures in question, since loading occurs constantly during normal (awake) life.

Abnormal biomechanical properties may have a number of causes, and some of those are clearly anatomical, such as avulsion. Hence, no firm conclusions can be drawn with regard to the treatment of this condition. However, we can conclude that interventions targeting vaginal tissue are likely to overlook one of the main pathophysiological factors, i.e. pelvic floor hyperdistensibility.

We found a statistically significant positive correlation between levator hiatal dimensions on maximum Valsalva and the degree of vaginal‐laxity symptom bother, which means that the larger the levator hiatus is, the greater the bother experienced from this symptom. Having said that, this correlation was weak, probably owing to the fact that the degree of symptom bother is confounded by a number of other factors such as, for example, the quality of any sexual relationship and the importance of coitus in the patient's sexual life.

Aydin *et al*.^15^ explored the association between levator hiatal biometry and female sexual function, assessed using the Female Sexual Function Index. Statistically significant differences in delta‐area and delta‐APD (delta being the difference between hiatal dimensions on maximum Valsalva and those at rest) of the levator hiatus were found between a low‐sexual‐function group and a normal‐sexual‐function group. No significant difference was found between the groups in measurements obtained at rest, nor those obtained on maximum Valsalva. The discrepancies between these results and ours can be explained by several factors. The study populations differed substantially, as the study of Aydin *et al*. included only patients asymptomatic for pelvic floor disorders, while our study population was recruited from a urogynecology clinic. Moreover, Aydin *et al*. investigated female sexual function in general, which involves not only physical, but also psychological, aspects. However, it is interesting that, despite differences in study design, both studies found a correlation with delta values of levator ani distensibility measurements, supporting the role of levator ani distensibility in female sexual function.

In another study, Thibault‐Gagnon *et al*.^16^ investigated the impact of childbirth‐related levator trauma on pelvic floor and sexual function using 4D‐TLUS and an in‐house validated questionnaire in women on average 5 months after childbirth. Interestingly, the presence of levator avulsion was correlated with a lower perception of pelvic floor muscle integrity and function, but levator ani overdistension was not. An explanation may be that the effect of levator avulsion is perceived earlier after delivery than is the effect of hyperdistensibility, as the patients were seen on average 5 months postpartum.

We acknowledge that this study has some strengths and weaknesses. A strength lies in the assessment of the sonographic parameters, which was performed blinded to all other data including symptoms and clinical findings. Another strength is its large population size. A major limitation is the composition of our study population, which consisted largely of Caucasian women recruited from a urogynecological clinic. This implies that our results may not be applicable to the general population. In our population, 93% of women in the vaginal‐laxity group also had significant prolapse, which is unlikely to be representative of the general population and can be explained by the fact that both conditions are associated with pelvic floor hyperdistensibility. Women with vaginal laxity without symptoms of prolapse may not seek medical help. However, if examined, we expect that a non‐negligible proportion of them would probably have early‐stage prolapse (not yet causing any typical prolapse symptoms) and an enlarged levator hiatal area on maximum Valsalva on TLUS. Another limitation of this study is its retrospective design and the fact that imaging data were obtained in clinical practice by multiple subspecialty trainees, albeit under the direct supervision of the senior author. However, this may also be considered as a strength rather than a weakness, given that it would tend to increase the general applicability of our results.

In conclusion, this retrospective study showed a statistically significant association between vaginal laxity and measures of levator ani hiatal distensibility obtained on maximum Valsalva maneuver, with levator hiatal area providing the highest predictive value.
